# Canary: an atomic pipeline for clinical amplicon assays

**DOI:** 10.1186/s12859-017-1950-z

**Published:** 2017-12-15

**Authors:** Kenneth D. Doig, Jason Ellul, Andrew Fellowes, Ella R. Thompson, Georgina Ryland, Piers Blombery, Anthony T. Papenfuss, Stephen B. Fox

**Affiliations:** 1 Research Division, Peter MacCallum Cancer Centre, East Melbourne, VIC Australia; 2 Department of Pathology, Peter MacCallum Cancer Centre, East Melbourne, VIC Australia; 3 Sir Peter MacCallum Department of Oncology, University of Melbourne, Melbourne, Australia; 4 Department of Pathology, University of Melbourne, Melbourne, Australia; 5 Department of Medical Biology, University of Melbourne, Melbourne, Australia; 6 Bioinformatics Division, The Walter and Eliza Hall Institute of Medical Research, Parkville, VIC Australia

**Keywords:** Targeted sequencing, Canary, PathOS, Pipelines, Clinical diagnostics, Variant calling, Amplicon

## Abstract

**Background:**

High throughput sequencing requires bioinformatics pipelines to process large volumes of data into meaningful variants that can be translated into a clinical report. These pipelines often suffer from a number of shortcomings: they lack robustness and have many components written in multiple languages, each with a variety of resource requirements. Pipeline components must be linked together with a workflow system to achieve the processing of FASTQ files through to a VCF file of variants. Crafting these pipelines requires considerable bioinformatics and IT skills beyond the reach of many clinical laboratories.

**Results:**

Here we present ***Canary***, a single program that can be run on a laptop, which takes FASTQ files from amplicon assays through to an annotated VCF file ready for clinical analysis. Canary can be installed and run with a single command using Docker containerization or run as a single JAR file on a wide range of platforms. Although it is a single utility, Canary performs all the functions present in more complex and unwieldy pipelines. All variants identified by Canary are 3′ shifted and represented in their most parsimonious form to provide a consistent nomenclature, irrespective of sequencing variation. Further, proximate in-phase variants are represented as a single HGVS ‘delins’ variant. This allows for correct nomenclature and consequences to be ascribed to complex multi-nucleotide polymorphisms (MNPs), which are otherwise difficult to represent and interpret. Variants can also be annotated with hundreds of attributes sourced from MyVariant.info to give up to date details on pathogenicity, population statistics and in-silico predictors.

**Conclusions:**

Canary has been used at the Peter MacCallum Cancer Centre in Melbourne for the last 2 years for the processing of clinical sequencing data. By encapsulating clinical features in a single, easily installed executable, Canary makes sequencing more accessible to all pathology laboratories.

Canary is available for download as source or a Docker image at https://github.com/PapenfussLab/Canary under a GPL-3.0 License.

**Electronic supplementary material:**

The online version of this article (doi:10.1186/s12859-017-1950-z) contains supplementary material, which is available to authorized users.

## Background

Clinical diagnostics is being transformed by technology capable of analysing patient DNA at the nucleotide level. The accuracy, turnaround time and reproducibility of clinical sequencing rely heavily on bioinformatics pipelines that convert raw sequencing data into meaningful variants. These pipelines are characterised by multiple software dependencies, lack of portability, complex parameter tuning and often need a cluster computing environment for parallel execution [1]. These attributes result in pipelines that are hard to deploy in a production clinical environment.

Here we introduce ***Canary***, a stand-alone Java utility that performs the function of multi-tool pipelines and can generate annotated VCF files directly from zipped FASTQ files generated from amplicon assays. As Canary only requires a Java runtime, it can be deployed on any computer with Java installed, in contrast to the myriad dependencies of most current pipelines. Additionally, it is available as a Docker [2] image from their public repository allowing it to be installed and run on any platform supporting Docker with a single command:% docker run -v /tmp/data:/canary.data dockercanary/canary

The processing of amplicon sequence data using ubiquitous shotgun sequencing pipelines leads to suboptimal results in terms of speed and quality [3, 4]. There are relatively few options for processing amplicon data outside of commercial platforms such as Illumina BaseSpace [5]. This platform only caters for proprietary Illumina assays such as Amplicon TruSeq and TruSight and does not allow for custom panels that target other genes or permit incorporating amplicon analysis into an in-house pipeline. Non-commercial amplicon software include: Mutascope [4] which doesn’t perform alignment, AmpliVar [6] which doesn’t perform variant calling and UNDR ROVER [3] which doesn’t normalise, 3’ shift or annotate the variants produced. Canary simplifies the pipeline steps required with a single command to go from zipped FASTQ files to an annotated VCF file suitable for clinical curation. It is assumed that the FASTQ files have been quality controlled previously by a program such as FASTQC [7].

To our knowledge, Canary is the only tool that can perform all the necessary pipeline steps of alignment, variant calling, normalisation, transcript selection and rich annotation in a single executable program.

### Amplicon Assays

**Fig. 1:**
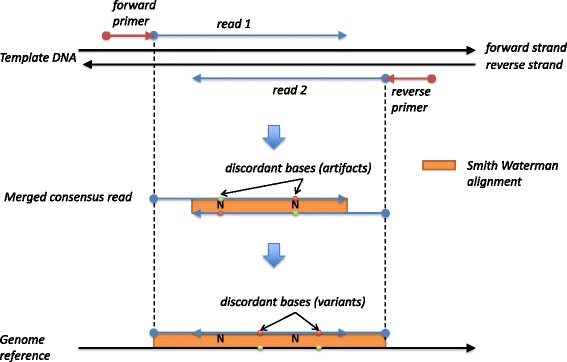
Canary read alignment. Overlapping amplicon reads are aligned to the reference genome in a two step process. The overlapping read pairs, that are derived from the same DNA molecule, are aligned to each other to form a single consensus merged read which is then aligned to a reference genome to identify variants

Targeted amplicon sequencing is a cost effective way for deeply sequencing a panel of genes of interest [8]. Its depth and ability to target specific gene regions, such as oncogene hot spots, makes it effective and efficient for cancer diagnostic assays. The design of amplicon assays calls for paired primers at specific genomic positions. As the position of these primers is known, this allows bypassing the computationally expensive alignment step of typical pipelines. Canary exploits this fact, along with read caching, to achieve rapid processing of FASTQ files and speeding up of overall sample turnaround time. If amplicon forward and reverse primers are separated by a distance slightly greater than sequencer read length, paired short reads will overlap the amplicon region of interest. Canary uses this overlap to combine paired reads and reduce random read errors. The combined read is then aligned to the reference amplicons to identify variants. Read combining and alignment is performed using a fast C++ Smith-Waterman library [9] (see Fig. 1). The targeted nature of amplicon assays reduces the volume of reads to process but allows for exact alignments without compromising speed. Additional speed is gained by implementing a read cache which hashes the combined, aligned reads along with any variant calls.

The advantage of amplicon panels is the targeted capture of specific regions of interest at very high coverage (>1000×) but with the disadvantage of a PCR step, which may amplify errors in poorly covered regions. This step masks copy number variation in the sample. To reduce false positive PCR artefacts, somatic samples can be sequenced as technical replicates and laboratories can report only variants appearing in both replicates. For all NA12878 control samples in 2017 sequencing runs (*n* = 133), the median percent of variants per sample that appeared in only one replicate was 38.8%. This represents a considerable workload saving but with the additional cost of reagents and wet lab processing.

In paired end sequencing, base quality decreases across the length of the reads and the second read is often of less quality than the first. These effects can be mitigated with amplicon sequencing by producing a consensus read from an overlapping amplicon read pair. The overall error rates are therefore discontinuous across the amplicon with lower rates in the overlapping region. Tiling multiple amplicons across regions of interest is an effective strategy for achieving high quality sequencing in critical areas of the genome.

### Variant Calling

To call variants, merged reads are aligned against the reference amplicons also using the Smith-Waterman algorithm. All variants found are cached in a hash map keyed by the raw read pair to speed up processing for recurring reads. Any variants occurring within 15 bp of each other (configurable) are also counted as compound in-phase variants as they occur within a merged read pair that is sequenced from a single strand of DNA. Variants further apart, but within the same merged pair are treated as independent events and counted as different variants. Although Canary reports MNPs, the individual component variants are also reported individually allowing the discretion of downstream reporting of either the MNPs or their constituent variants. For example, the variant BRAF:c.1798_1799delinsAG p.V600R would also be reported as BRAF:c.1798G > A p.V600 M and BRAF:c.1799 T > G p.V600G. It is noteworthy that the latter two variants individually predict different amino acids (Methionine and Glycine) while the ‘delins’ variant predicts Arginine highlighting the need for reporting MNPs to correctly identify molecular consequences.

After all reads have been processed, variant counts are summed and if they exceed the minimum read count and variant allele frequency (VAF) thresholds, they are passed to the normalisation phase.

The use of the Smith-Waterman alignment of merged reads against the amplicon reference sequence allows for large indels to be accurately called without a computationally expensive indel realignment step. The maximum size deletion called for all sequence results, in 2016, was 191 bp (median = 1 bp, *n* = 190,552 variants) and the maximum insertion was 78 bp (median = 1 bp, *n* = 73,107 variants).

### Variant Normalisation

In clinical variant reporting, it is routine to use the Human Genome Variation Society (HGVS) nomenclature [10], with explicitly named Refseq transcripts [11]. Pipelines and variant callers produce VCF files of variants described with [chromosome, position, reference base, alternate base] tuples. This representation is unsuitable for clinical use as multiple tuples can map to a single genomic change. Due to the difficulty of representing larger indels and MNPs, many laboratories may report and submit variants to curation databases in a non-canonicalised or incorrect notation.

Canary is able to correctly render variants, together with a Refseq transcript, as HGVSg, HGVSc and HGVSp in their most parsimonious 3′ shifted form [12]. Complex in-phase multi-nucleotide variants are correctly rendered as ‘delins’ variants saving error-prone manual interpretation - a common cause of clinical variant description error. By performing these complex operations, Canary allows downstream curation and reporting systems to consistently and correctly report clinical variants. Variants are normalised in Canary by multiple queries to the Mutalyzer [13] web site API, which can be locally installed on a virtual machine for convenience. VCF variants are converted to HGVSg format and batch submitted and converted to one or more (Refseq transcript, HGVSc, HGVSp) tuples. For variants with multiple transcripts, the preferred tuple is chosen from a gene to transcript list, which maps a gene to the single most ‘common’ transcript for a gene. An editable gene to transcript mapping file is provided with Canary. This list has been manually curated by the Peter MacCallum Cancer Centre but other laboratories may prefer different transcripts for reporting. It has been shown that the choice of transcript can significantly impact variant annotation [14].

If multiple preferred transcripts are found for a variant, the transcript with the variant closest to an exon is chosen. This transcript is then batch validated with the Mutalyzer API. Variants are 3′ shifted with respect to the chosen transcript and, if needed, any ‘ins’ variants are changed to their ‘dup’ equivalent if necessary. Any variants that are 3′ shifted are then resubmitted back to the API to shift their chromosome position as well. The final genomic position will match the cDNA position for both positive and negative strand transcripts. Although not strictly matching the rules for HGVSg variants (where variants are right shifted with respect to the chromosome) this approach is felt to be less confusing.

**Fig. 2:**
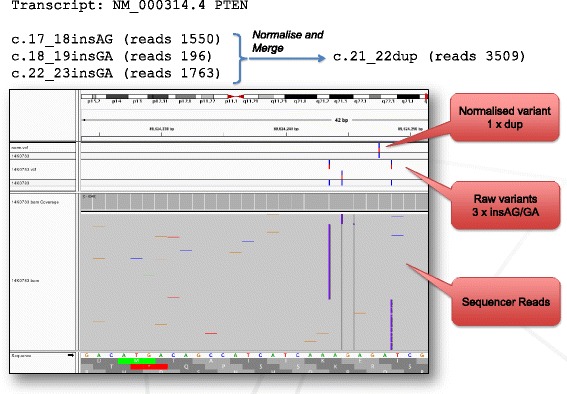
Normalised variants displayed in IGV. IGV display of Illumina MiSeq reads from a clinical patient highlighting the variation in the representation of indels within BAM files. The same variant is represented differently in three sets of reads which need to be merged to a single locus with the standardized HGVS nomenclature of NM_000314.4:c.21_22dup. Additionally, the reads contributing to the three read sets must be combined to calculate the correct variant allele frequency

The above process ensures a unique representation for each variant and allows for consistent matching between sequencing runs, patients and databases. See Fig. 2.

### Annotation

Accessing and maintaining comprehensive and timely annotation data sources is an onerous task for any laboratory but this is critical in a clinical setting. Common tools for annotating VCF files and other genomic information include; Annovar [15, 16], SnpEff [17] and Variant Effect Predictor (VEP) [18]. These tools have, until recently, required the downloading of large datafiles or caches to operate and places the burden of large data storage and maintenance on the users. The increasing emergence of API driven annotation improves the timeliness of data and reduces the overhead of accessing the latest genomic annotations. Canary harnesses this trend by using online sites for both genomic transcript nomenclature (Mutalyzer.nl [13]) and variant annotation (MyVariant.info [19]). Using online data sources avoids the need to download very large databases of gene, transcript and variant information, which must be managed and refreshed periodically to maintain currency. These two online sources are maintained by groups that specialise in keeping the data current while also aggregating reference data sources from NCBI, Clinvar, dbNSFP, CADD and many others.

If supplied with a list of MyVariant data fields, Canary will retrieve these annotations for each variant and add these to INFO field of the generated VCF file. These annotations are retrieved in real-time from MyVariant as the file is created and cover most of the useful annotation sources available [20]. Fields are described in a hierarchical “dotted” notation, which indicates the underlying data source, for example, the gene protein domain from Cadd [21] data is specified as cadd.gene.prot.domain. A list of desired annotations can be passed as a file to Canary, which will add this data to the VCF file. Note that not every variant will have every annotation attribute as some data will not be relevant or available for every variant.

### Usage

A typical command line usage is shown below together with explanations of options. More detailed descriptions and additional command line options may be found in Additional file 1. The code provides additional entry points if the functions need to be embedded within a larger system.

Canary can be also used as a utility within a larger pipeline to just annotate a VCF file by enriching its ‘INFO’ fields with correct HGVS nomenclature and optionally generating a tab separated variant file suitable for directly loading into a spreadsheet or uploading to a database.

### Results

To assess the performance of Canary in both germline and somatic contexts, three experiments were performed with well-studied samples containing known variants.

To evaluate performance with germline samples, DNA from the Coriell cell line DNA NA12878 (Coriell Cell Repository, Camden, NJ) was sequenced in 54 runs over a period of eight months during 2017 as a control sample on a custom myeloid amplicon panel on Illumina MiSeq instruments. Two NA12878 samples were sequenced as technical replicates on each run giving a total of 108 samples sequenced. Nine NA12878 high confidence variants were found to intersect the amplicon capture region by using bedtools v2.26 [22] and the NIST Genome in a Bottle NA12878 vcf/bed file repository [23]. The sequencer generated FASTQ files were used by Canary to create annotated VCF files calling variants with greater than 20% variant allele frequency. Of the total true positives (TP) expected, 972 = 54 * 2 * 9, Canary found 970. The FASTQ files for the missing two variants were inspected. One contained reduced coverage for the amplicon at the locus where the true positive should have been but contained the variant at a VAF = 1.95% which was below the Canary VAF threshold setting. The other FASTQ file contained sufficient reads but no true positive variant. It did however, contain a novel variant (for the control) suggesting wet lab contamination. If we exclude these two samples as likely wet lab issues, Canary recovered 100% of the control variants. An additional 1124 false positive (FP) variants were called giving a precision (TP/(TP + FP)) of 46.3% and a sensitivity (TP/(TP + FN)) of 100%. Of these false positives, 84.9% were variants occurring in more than 35% of samples (including patient samples and NA12878 control samples) analysed by the assay during this period. Because of their high frequency in the assay results, they are inferred to be technical artefacts due to amplicon primer specific artefacts. For routine clinical assays, these recurrently occurring assay variants are flagged by the pipeline and excluded from further analysis.

To compare Canary to other pipelines, three pipelines were run on ten NA12878 samples sequenced in ten runs during June and July 2017. The three pipelines used were; 1) Canary performing both read alignment and variant calling down to a variant allele frequency of 20%, 2) BWA-MEM 2 (0.7.20) performing read alignment and GATK haplotype caller for variant calling and 3) BWA-MEM 2 performing read alignment and VarDict for variant calling. The summary results, including raw calls, TP, FP, FN, sensitivity and precision, are shown in Additional file 2: Table S1. All pipelines recovered all nine true positive variants except for one variant not found by VarDict. Canary was shown to have the best precision with Canary 42.8%, GATK 21.9%, VarDict 17.1%, Sensitivity: Canary 100%, GATK 100%, VarDict 98.9%). For detailed results see Additional file 2: Table S1 in the supplementary files.

**Fig. 3:**
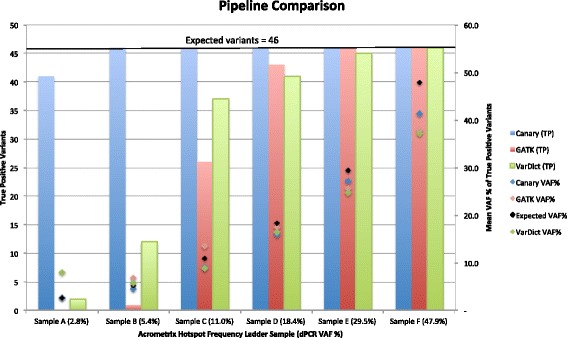
Comparison of Canary with BWA, GATK and VarDict. Graph showing the number of true positive (TP) variants (expected = 46) for three pipelines run against six Acrometrix control samples containing known variants at a certified allele frequency (left hand axis). The three pipelines were; Canary performing read alignment and variant calling (blue bars), BWA-MEM 2 performing read alignment and GATK haplotype caller for variant calling (red bars) and BWA-MEM 2 performing read alignment and VarDict for variant calling (green bars). The mean variant allele frequency for each of the pipeline variants is shown as coloured diamonds and the control sample expected frequency is shown as black diamonds (right hand axis). Raw data and statistics are available in Additional file 2: Table S1

To evaluate somatic performance, samples from the Acrometrix Oncology Hotspot Frequency Ladder were used (Thermofisher, Australia) [24]. The ladder samples consist of a synthetic “chromosome” mixed with genomic DNA from the Personal Genome Project cell line GM24385 [25]. The synthetic variants were confirmed by Sanger sequencing and the frequencies in each of six dilutions were quantified by digital droplet PCR. The frequencies provided were 48%, 29%, 18%, 11%, 5%, and 3%. The samples were run on an Illumina MiSeq sequencer and the reads converted to paired-end FASTQ files. These files were then run against the three pipelines described above except that the Canary pipeline called variants down to a variant allele frequency of 1%. The pipelines were run on the Acrometrix samples to generate VCF files and determine true positives (TP) *n* = 46, false positives (FP) and false negatives (FN). Variant read depth was also used to compare against the expected allele frequencies of the 46 expected variants. See Fig. 3 and Additional file 2: Table S1. Instances of the pipeline commands and their parameters can be found in Additional file 3: File 3. All pipelines performed acceptably in recapitulating the VAF of the samples with Canary performing best (Mean VAF differences; Canary 2.3%, GATK 3.6%, VarDict 4.2%). Canary showed the best sensitivity and fully recovered all expected variants at all allele frequencies except for five variants in Sample A with the lowest expected VAF of 2.8%. In contrast, the GATK and VarDict pipelines performed increasingly poorly at low allele frequencies and showed both lower average sensitivity and lower average precision than Canary. (Sensitivity: Canary 98.2%, GATK 58.7%, VarDict 66.3%, Precision: Canary 7.5%, GATK 1.0%, VarDict 0.9%). These results would not be representative of normal practice, as pipeline parameters will usually be tuned for their corresponding assay. It would also be more common to use GATK MuTect for a tumour/normal somatic assay but normal samples were not available for these samples. We also note that VarDict also supports an amplicon mode and a tumour-normal mode in addition to its default single sample non-amplicon mode. When run on the Acrometrix samples in amplicon mode, VarDict performed more poorly (fewer true positive variants) than in its default mode.

Canary was also faster than the other pipelines over three repeated runs of Sample F with the following mean wall clock run times, Canary 14.0 min., GATK 16.3 min. and VarDict 32.0 min. These runs were performed on a heterogeneous cluster with comparable loads for all runs. Both the GATK and VarDict pipelines used multithreading to achieve faster throughput but Canary currently only supports single thread execution. Execution is currently proportional to the number of amplicons times the number of reads and would lend itself to parallel execution in subsequent releases. The GATK and VarDict times do not include annotation times for these pipelines whereas annotation is built into the Canary processing allowing its output to be readily incorporated into downstream workflows. Canary was also run three times on Sample F on a Mac laptop with 16Gb of memory with a mean elapsed runtime of 14.0 min, matching cluster runtimes.

Typical performance of Canary is between 7 and 10 min when processing a full Illumina MiSeq run of 48 samples (22 patient samples in replicate and 4 controls), performing alignment, variant calling and annotation, on a computing cluster. These times are for an in-house myeloid assay of 216 amplicons covering key exons of 26 genes with a total panel size of 29.9 kilobases. The average read pairs per sample were 375,522 and the average cache hit ratio was 20.5%.

### Conclusion

Canary has been used with custom and commercial amplicon diagnostic panels as part of PathOS, a complete clinical diagnostic system [26]. It is integrated into clinical pipelines within the Molecular Pathology Department of the Peter MacCallum Cancer Centre for over a year while the Normalisation module has been used for the last two years.

Since its introduction into the diagnostic pipeline in November 2015, Canary has processed 8,203 patient samples and identified 199,693 variants of which 5,055 were clinically reportable in 1,880 patients. The reported variants included 498 deletions, 78 insertions and 76 MNPs. The MNPs comprised variants with a combined deletions of between 1 and 28 bp and insertions of between 1 and 11 bp.

Canary has also been integrated into a Minimum Residual Disease (MRD) pipeline used to detect recurrent indels due to disease relapse in haematological malignancies post therapy (manuscript accepted for publication). This pipeline employs ultra-deep (500,000×) sequencing to detect a single read or more containing the recurrence of an indel originally detected in the patient sample prior to therapy. Indels used for MRD are sufficiently unlikely to occur as the result of random error.

In conclusion, Canary fills the need for a readily deployable amplicon pipeline utility capable of rendering complex variants with consistent and correct nomenclature suitable for clinical reporting. Available as a Docker image, it is easily integrated into laboratories needing to perform the necessary pipeline steps of alignment, variant calling, normalisation, transcript selection and rich annotation within a single executable program.

### Availability

Project Name: Canary.

Project Home Page: https://github.com/PapenfussLab/Canary

Operating System(s): Docker [2] compatible OS (e.g. Linux, Mac, AWS, Azure, Windows).

Programming Languages: Groovy [27], Java.

Other requirements: Reference data.

License: GNU license - GPL 3.0 [28].

## Additional files

Additional file 1: canary usage.docx: Description of the Canary command line options. (DOCX 136 kb)

Additional file 2: Table S1. xlsx: Comparison statistics for Canary, BWA, GATK and VarDict. (XLSX 45 kb)

Additional file 3: File 3. Example Pipeline Commands.txt: Validation pipeline command examples. (TXT 6 kb)

## Abbreviations

API: Application Programming Interface
BAM: Binary Alignment Map format
CADD: Combined Annotation Dependent Depletion
cDNA: complementary DNA
delins: A variant which combines a deletion and an insertion
FN: False negatives
FP: False positives
HGVS: Human Genome Variant Society
indel: Insertion / Deletion
MNP: Multi-nucleotide polymorphism
MRD: Minimum Residual Disease
NATA: National Association of Testing Authorities
NGS: Next Generation Sequencing
NGS: Next Generation Sequencing
PCR: Polymerase Chain Reaction
TP: True positives
TSV: Tab separated variable format
VAF: Variant Allele Frequency
VCF: Variant Call Format
