# Supplementary material for: Canary: an atomic pipeline for clinical amplicon assays
Source: BMC Bioinformatics. 2017 Dec 15;18:555. doi: 10.1186/s12859-017-1950-z (PMC5732437; doi:10.1186/s12859-017-1950-z)
Supplement: Supplementary file 1 — canary usage.docx: Description of the Canary command line options. (DOCX 136 kb) [file 12859_2017_1950_MOESM1_ESM.docx]

usage: Canary [options] read1.fastq.gz read2.fastq.gz

| Short Option | Long Option | Argument Type | Description |
| --- | --- | --- | --- |
| -a | --amplicon | FASTA file (input) | Amplicon FASTA file [required] |
| -ano | --annotation | Text file (input) | File of MyVariant annotation fields |
| -b | --bam | BAM file (output) | Optional BAM file of alignment |
| -c | --complex |  | Coalesce complex events aka MNPs |
| -cols | --columns | Text file (input) | File of VCF field names to output to TSV (one per line with optional alias after comma) |
| -d | --debug |  | Turn on debugging (Note: will generate large file of alignments [debug.out]) |
| -fastq | --fastq | File prefix (output) | Optional FASTQ output files prefix |
| -filt | --filter | List | List of comma separated amplicon names to use |
| -h | --help |  | Output a help message |
| -maxmut | --maxmut | Integer | Maximum number of mutations allowed per read pair [10] |
| -minpair | --minpair | Integer | Min read pairs for variants [10] |
| -mingap | --mingap | Integer | Maximum size of inter mutation gap for complex mutations [15] |
| -mnpmax | --mnpmax | Integer | Maximum size of complex mutations [30] |
| -mut | --mutalyzer | URL | Mutalyzer annotation server host [https://mutalyzer.nl] |
| -n | --nocache |  | Don’t use read cache |
| -norm | --normalise |  | Generates annotated VCF file from VCF output |
| -o | --output | Filename (output) | Output report file |
| -p | --primers | Filename (input) | Amplicon Primers file [required] |
| -r | --reads | Number | Per cent of reads to process [100] |
| -t | --tsv | TSV File (output) | TSV (Tab separated variable) file of VCF output |
| -ts | --transcript | File (input) | File of transcripts mapping genes -> Refseq (without version number) |
| -v | --vcf | VCF file (output) | Found variants VCF file [canary.vcf] |
| -vaf |  | Number | Minimum VAF for variants [1.0%] |
| -ver | --version |  | Display Canary version and exit |
